# Related factors of the nursing diagnosis ineffective breathing pattern in an intensive care unit*

**DOI:** 10.1590/1518-8345.2902.3153

**Published:** 2019-10-07

**Authors:** Patricia Rezende do Prado, Ana Rita de Cássia Bettencourt, Juliana de Lima Lopes

**Affiliations:** 1 Universidade Federal do Acre, Rio Branco, AC, Brasil.; 2 Bolsista da Coordenação de Aperfeiçoamento de Pessoal de Nível Superior (CAPES), Brasil,; e da Fundação de Amparo à Pesquisa do Acre (FAPAC), Brasil.; 3 Universidade Federal de São Paulo, Escola Paulista de Enfermagem, São Paulo, SP, Brasil.

**Keywords:** Risk Factors, Signs and Symptoms, Nursing Diagnosis, Respiratory System, Classification, Nursing, Fatores de Risco, Sinais e Sintomas, Diagnóstico de Enfermagem, Sistema Respiratório, Classificação, Enfermagem, Factores de Riesgo, Signos y Síntomas, Diagnóstico de Enfermería, Sistema Respiratório, Clasificación, Enfermería

## Abstract

**Objective:**

to identify the predicting factors and sensitivity, specificity, positive and negative related value of nursing diagnosis Ineffective Breathing Pattern among patients of an intensive care unit.

**Method:**

cross-sectional study. A logistic regression was fitted to assess the simultaneous effects of related factors.

**Results:**

among the 120 patients, 67.5% presented Ineffective Breathing Pattern. In the univariate analysis, the related factors were: group of diseases, fatigue, obesity and presence of bronchial secretion, and the defining characteristics were: changes in respiratory depth, auscultation with adventitious sounds, dyspnea, reduced vesicular murmurs, tachypnea, cough and use of the accessory musculature to breathe. The mean age of patients with was higher than those without this diagnosis. The defining characteristics reduced murmurs had high sensitivity (92.6%), specificity (97.4%), negative related value (86.4%) and positive related value (98.7%). The related factors of Ineffective Breathing Pattern were the related factors fatigue, age and group of diseases.

**Conclusion:**

fatigue, age and patients with a group of diseases were related factors of Ineffective Breathing Pattern in this study. Reduced vesicular murmurs, auscultation with adventitious sounds and cough may be defining characteristics to be added in the international classification, as well as the related factors bronchial secretion and group of diseases.

## Abstract (pt)

**Objetivo:**

identificar os fatores preditores e a sensibilidade, especificidade, valores preditivos positivo e negativo do diagnóstico de enfermagem Padrão respiratório ineficaz em pacientes de uma unidade de terapia intensiva.

**Método:**

estudo transversal. Foi realizada a regressão logística para avaliar os efeitos simultâneos dos fatores relacionados.

**Resultados:**

dos 120 pacientes, 67,5% apresentaram padrão respiratório ineficaz. Na análise univariada, os fatores relacionados foram: grupo de doenças, fadiga, obesidade e a secreção nos brônquios, e as características definidoras foram: alteração na profundidade respiratória, ausculta com ruídos adventícios, dispneia, murmúrios vesiculares diminuídos, taquipneia, tosse e uso da musculatura acessória para respirar. A média de idade dos pacientes com este diagnóstico foi superior ao daqueles sem este diagnóstico. A característica definidora murmúrios vesiculares diminuídos teve alta sensibilidade (92,6%), especificidade (97,4%), valor preditivo negativo (86,4%) e valor preditivo positivo (98,7%). Os fatores relacionados de padrão respiratório ineficaz foram a fadiga, a idade e o grupo de doenças.

**Conclusão:**

a fadiga, a idade e pacientes com grupo de doenças foram fatores preditores do diagnóstico padrão respiratório ineficaz neste estudo. Os murmúrios vesiculares diminuídos, a ausculta com ruídos adventícios e a tosse podem ser características definidoras a serem acrescentadas na classificação internacional, assim como os fatores relacionados secreção nos brônquios e grupo de doenças.

## Abstract (es)

**Objetivo:**

identificar los factores predictores y la sensibilidad, especificidad, valores predictivos positivo y negativo del diagnóstico de enfermería padrón respiratorio ineficaz en pacientes de una unidad de terapia intensiva.

**Método:**

estudio transversal. Se ajustó una regresión logística para evaluar los efectos simultáneos de factores relacionados.

**Resultados:**

de los 120 pacientes, 67,5% presentaron padrón respiratorio ineficaz. En el análisis univariado, los factores relacionados que se asociaron con este diagnóstico fueron: grupo de enfermedades, fatiga, obesidad y la secreción en los bronquios y las características definitorias que se asociaron fueron alteración en la profundidad respiratoria, ausculta con ruidos adventicios, disnea, murmullo vesicular disminuido, taquipnea, tos y uso de la musculatura accesoria para respirar. El promedio de edad de los pacientes con padrón respiratorio ineficaz fue superior al de aquellos sin padrón respiratorio ineficaz. Las características definitorias redujeron los soplos con alta sensibilidad (92.6%), especificidad (97.4%), valor negativo relacionado (86.4%) y valor positivo relacionado (98.7%). Los factores relacionados con el patrón de respiración ineficaz fueron los factores relacionados con la fatiga, la edad y el grupo de enfermedades.

**Conclusión:**

la fatiga, la edad y los pacientes con grupo de enfermedades relacionadas con el trauma, cardiocirculatorio y otros grupos de enfermedades fueron factores predictores del diagnóstico padrón respiratorio ineficaz en esta unidad. Los murmullos vesiculares disminuidos, la auscultación con ruidos adventicios y la tos pueden ser características definitorias a ser añadidas en la clasificación internacional, así como los factores relacionados de secreción en los bronquios y grupo de enfermedades.

## Introduction

The evaluation of the breathing pattern is essential to define nursing interventions and care plan to meet the patients’ needs. This evaluation is performed through a physical examination, monitoring the physiological functions of chest examination, palpation, pulmonary percussion and auscultation, which provide objective data on the use of respiratory muscles, respiratory rate and lung sounds^1-2^.

In normal conditions, the breathing pattern satisfies the need for oxygenation of the body. However, situations where there is fatigue, airway impairment due to secretion and decreased pulmonary expansion characterize the nursing diagnosis (ND) ineffective breathing pattern (IBP)^3^.

The ND ineffective breathing pattern (IBP) was first defined in 1980 and revised in 1996, 1998, 2010 and 2017. In 2017, this diagnosis was modified and included associated conditions. This diagnosis focuses on problem and belongs to domain 4, class 4, activity/rest of the NANDA International, Inc. (NANDA-I). IBP is defined as an inspiration and/or expiration pattern that does not provide sufficient ventilation^4^.

This diagnosis has often been identified in adult individuals and in several units. In trauma patients treated at an university hospital in the city of São Paulo, Brazil, it was observed that 82.4% presented IBP^5^ and 85.7% in adults who receive care in emergency rooms^6^. In adult patients with heart disease, it was observed that this diagnosis was present in 70.6% and that 100.0% of them presented fatigue as a related factor (RF) and dyspnea as a defining characteristic (DC)^7^.

The first American survey identified the Nursing Diagnosis IBP in 81.0% of intensive care patients^8^. In the city of Rio Branco, Acre (AC), Brazil, a prevalence of 64.4% of IBP was identified in an Intensive Care Unit (ICU) patients^9^ however, these studies evaluated only the prevalence, did not identify the measures of accuracy and also did not evaluate the predicting factors of the IBP nursing diagnosis. Thus, it is observed that the nursing diagnosis IBP is very frequent in ICUs. Due to the importance of early identification and the establishment of a care plan for these patients, the objective of the present study was to identify the predicting factors and sensitivity, specificity, positive and negative related value of nursing diagnosis Ineffective Breathing Pattern among patients of an intensive care unit.

## Method

This is an observational, cross-sectional, analytical study. The research was performed at the ICU of the Urgency and Emergency Hospital of the city of Rio Branco, AC, Brazil, from September 2015 to April 2016. The sample size was calculated by the formula for finite populations, using a 95% confidence coefficient; a random error of 5%; IBP prevalence of 64.4%, according to a study carried out at an ICU of the city of Rio Branco^9^, and the population of 180 individuals, according to the number of conscious and oriented patients hospitalized at this unit, over a period of one year. This profile of patients considered the need to perform the manovacuometry test. Thus, the sample size was 109 patients. Considering a 10% probability of loss, data were collected from 120 patients.

The inclusion criteria were: adult patients over 18 years of age, conscious and oriented, without neuromuscular disease identified by the medical and nurse evaluation and recorded in the medical record, with spontaneous breathing, which accepted and were able to undergo the manovacuometry test. Patients with hemodynamic instability that could interfere with the manovacuometry test were excluded^10-11^.

The independent study variables (DC, RF and associated conditions) were identified in the NANDA-I, classification for the Nursing diagnosis IBP^4^ and in a literature review (age, smoking, group of diseases, bronchial secretion, cough, reduce vesicular murmurs and auscultation with adventitious sounds)^12^. These selected variables were evaluated only by the main investigator of the study through interviews and physical examinations in the patients’ bed, according to the conceptual and operational definition of each variable, in which some were previously validated^13-15^and others were adapted for the adult population, such as assumption of three-point position to breathe, bradypnea/tachypnea values. The conceptual and operational definitions of the variables that have not been validated in other studies were elaborated by the researchers, according to the literature^2,16-21^.

All DC and RF were categorized as present or absent only by the main investigator, except for the group of diseases that were categorized according to the group diagnosed by the physician. The main groups of diseases identified were trauma (wound caused by gunshot and melee weapon, femur fracture and traumatic brain injury); cardiocirculatory diseases (acute myocardial infarction, atrioventricular block and ischemic or hemorrhagic stroke); diseases of the respiratory system (acute pulmonary edema and pneumonia); and other groups of diseases (acute abdomen, sepsis, snakebite, drowning, convulsion, exogenous intoxication, exploratory laparotomy, systemic lupus erythematosus, leptospirosis, pancreatitis and pregnancy-specific hypertension).

The defining characteristics of IBP evaluated were: changes in respiratory depth when assuming a three-point position to breathe, nose wing beats, bradypnea, increased anteroposterior chest diameter, decreased inspiratory pressure, decreased expiratory pressure, dyspnea, altered chest excursion, prolonged expiratory phase, orthopnea, abnormal breathing pattern, pursed-lip breathing, tachypnea, use of accessory muscles to breathe, decreased minute ventilation, cough^12^, auscultation with adventitious sounds^12^, and reduced vesicular murmurs^4,12^.

The related factors of IBP evaluated were: anxiety, pain, fatigue, respiratory muscle fatigue, hyperventilation, obesity, position of the body that prevents lung expansion, and bronchial secretion^4,12^. The RF age, smoking and a group of diseases were also included^12^.

The associated conditions of IBP evaluated were: chest wall deformity, bone deformity, musculoskeletal damage and hypoventilation syndrome^4^The associated conditions neurological damage, neurological immaturity, spinal cord injury and neuromuscular dysfunction were excluded because in these situations’ patients could not undergo the manovacuometry and the impossibility to perform this test was an exclusion criterion in the present study. The DC decreased vital capacity was not evaluated because there was no ventilator or spirometer in the unit, hindering the evaluation of the maximum percentage level of exhaled gas after maximal inspiration. These were limiting factor of the study. The outcome variable (dependent) studied was the presence of the nursing diagnosis IBP, defined as “inspiration and/or expiration that does not provide adequate ventilation”^4^. To have this diagnosis, patients should have three or more DC and maximal inspiratory pressure less than 80 cmH_2_O for men and less than 60 cmH_2_O for women^22^. The maximum inspiratory pressure was obtained through manovacuometry^10^ and is a simple way to measure maximum respiratory pressures, and a quantitative measure of respiratory muscle function and strength, which indicates if ventilation is adequate.

The DC decreased inspiratory pressure evaluated by the manovacuometer was chosen to confirm the presence of IBP because a study conducted in 2015 and 2016 with 626 adult ICU patients showed that this DC and the RF fatigue were the ones that had the greatest sensitivity for the IBP diagnosis in these patients^23^.

The nurse responsible for this research collected the data with aid of a standardized collection instrument prepared for this purpose. Collection was performed every day in the morning with patients who met the inclusion criteria in the ICU. After the data collection, the patients presenting and not presenting the IBP nursing diagnosis were compared to the causality of IBP nursing diagnosis.

For manovacuometry, the subjects were evaluated in the seated position (90º), using a nasal clip and semi-rigid rubber, diver type, with a internal hole of 2 mm diameter, in which the patient was asked to seal his lips firmly around the mouthpiece. In order to measure the maximal inspiratory pressure (MIP), the patient was asked to exhale, and at that moment the researcher occluded the orifice of the device and then the patient made a maximal inspiratory effort against the occluded airway, which was recorded on the manovacuometer. Patients would maintain the inspiratory pressure for at least 1.5 seconds and the highest sustained negative pressure was recorded. This same process was repeated three times, with one-minute intervals in each evaluation, and only the highest value was used^11^. It is emphasized that if differences of values greater than 10% were obtained between measurements, they were discarded.

The manovacuometer used was for single-use, of analog type, Wika manufacturer, model 611.10.063L, +120/-120, calibrated according to the internal procedure PRP-04-re.13, from NBR-ISO-10012, part 1. After the evaluation, the patients were allocated into two groups, with and without IBP.

This project was submitted to the Research Ethics Committee (REC) of the Federal University of São Paulo (UNIFESP) and approved under Opinion n^o^ 1,290,590, CAAE: 39185814.9.0000.5505, 21/10/2015. This research followed the guidelines of the Resolution of the National Council of Ethics in Research (CONEP) nº 466/2012, of the National Health Council (NHC) of Brazil and all the patients who were interviewed were informed and signed an Informed Consent Term authorizing the research.

Data were analyzed using the *Statistical Package for Social Sciences* (SPSS), Microsoft Office, University of Chicago, version 20.0. An initial descriptive analysis of the data was performed. Absolute and relative frequencies were used for the categorical variables and summary measures (mean, quartiles, minimum, maximum, and standard deviation) were used for the numerical variables.

The association between two categorical variables was verified using the Chi-square test, or the Fisher’s exact test in cases of small samples. When differences were observed in the distributions, standardized adjusted residues were used to identify local differences. Comparison of means between two groups was performed using Student’s t-test for independent samples.

For all defining characteristics and related factors of dichotomous nature, accuracy measurements were presented through sensitivity, specificity, positive related value (PPV) and negative related value (NPV). Logistic regressions were fitted to evaluate the simultaneous effects of RF on the presence of IBP. Due to the large number of variables that predicted the size of the sample, the variables whose associations with the dependent variable were significant at 20% in the univariate analysis were selected for the initial models. Then the non-significant variables at 5% were excluded one by one in order of significance (*backward* method).

The Hosmer and Lemes how test was used to analyze the goodness of fit of the final model, considering the RF as related variables. Sensitivity and specificity were calculated based on the ROC curve, which allowed the definition of a cutoff point in the probabilities of occurrence of IBP estimated from the adjusted final regression model. A significance level of 5% was used for all statistical tests.

## Results

From the 120 patients in the sample, 30.0% were elderly, with a mean age of 47 years, 60.8% were males, 59.2% were brown and 61.7% had primary schooling. The main groups of diseases identified were trauma (wound caused by gunshot and melee weapon, femur fracture and traumatic brain injury); cardiocirculatory diseases (acute myocardial infarction, atrioventricular block and ischemic or hemorrhagic stroke); diseases of the respiratory system (acute pulmonary edema and pneumonia); and other groups of diseases (acute abdomen, sepsis, snakebite, drowning, convulsion, exogenous intoxication, exploratory laparotomy, systemic lupus erythematosus, leptospirosis, pancreatitis and pregnancy-specific hypertension).

Among the evaluated patients, 67.5% presented the nursing diagnosis IBP. The Table 1 shows that IBP was associated with the following DC: changes in respiratory depth, auscultation with adventitious sounds, dyspnea, reduced vesicular murmurs, tachypnea, cough and use of accessory muscles to breathe. It was noted that 100% of the patients with these DC, except reduced vesicular murmurs, presented IBP.

**Table 1:** Defining characteristics according to the presence or absence of the Nursing diagnosis Ineffective Breathing Pattern. Rio Branco, AC, Brazil, 2015-2016

	**Ineffective Breathing Pattern**				**Total**		**ODDS RATIO**	p-value*

	**Absent**		**Present**					

	**n**	**%**	**n**	**%**	**n**	**%**		
Changes in respiratory depth	39	32.5%	81	67.5%	120	100.0%		<0.001*
Absent	39	44.8%	48	55.2%	87	100.0%	1.00	
Present	0	0.0%	33	100.0%	33	100.0%	(1)^†^	
Auscultation with adventitious sounds	39	32.5%	81	67.5%	120	100.0%		<0.001*
Absent	39	62.9%	23	37.1%	62	100.0%	1.00	
Present	0	0.0%	58	100.0%	58	100.0%	(1)^†^	
Nose wing beats	39	32.5%	81	67.5%	120	100.0%		0.172^‡^
Absent	39	33.9%	76	66.1%	115	100.0%	1.00	
Present	0	0.0%	5	100.0%	5	100.0%	(1)^†^	
Bradypnea	39	32.5%	81	67.5%	120	100.0%		-
Absent	39	32.5%	81	67.5%	120	100.0%	-	
Increased anteroposterior chest diameter	39	32.5%	81	67.5%	120	100.0%		0.172^‡^
Absent	39	33.9%	76	66.1%	115	100.0%	1.00	
Present	0	0.0%	5	100.0%	5	100.0%	(1)^†^	
Dyspnea	39	32.5%	81	67.5%	120	100.0%		<0.001*
Absent	39	41.9%	54	58.1%	93	100.0%	1.00	
Present	0	0.0%	27	100.0%	27	100.0%	(1)^†^	
Altered chest excursion	39	32.5%	81	67.5%	120	100.0%		0.052^‡^
Absent	39	34.8%	73	65.2%	112	100.0%	1.00	
Present	0	0.0%	8	100.0%	8	100.0%	(1)^†^	
Abnormal breathing pattern	39	32.5%	81	67.5%	120	100.0%		0.328^‡^
Absent	3	60.0%	2	40.0%	5	100.0%	1.00	
Present	36	31.3%	79	68.7%	115	100.0%	3.29	
Prolonged expiratory phase	39	32.5%	81	67.5%	120	100.0%		1.000^‡^
Absent	39	32.8%	80	67.2%	119	100.0%	1.00	
Present	0	0.0%	1	100.0%	1	100.0%	(1)^†^	
Pursed-lip breathing	39	32.5%	81	67.5%	120	100.0%		1.000^‡^
Absent	39	33.1%	79	66.9%	118	100.0%	1.00	
Present	0	0.0%	2	100.0%	2	100.0%	(1)^†^	
Reduced vesicular murmurs	39	32.5%	81	67.5%	120	100.0%		<0.001*
Absent	38	86.4%	6	13.6%	44	100.0%	1.00	
Present	1	1.3%	75	98.7%	76	100.0%	475.00	
Orthopnea	39	32.5%	81	67.5%	120	100.0%		1.000^‡^
Absent	39	32.8%	80	67.2%	119	100.0%	1.00	
Present	0	0.0%	1	100.0%	1	100.0%	(1)^†^	
Decreased expiratory pressure	39	32.5%	81	67.5%	120	100.0%		-
Present	39	32.5%	81	67.5%	120	100.0%	-	
Decreased inspiratory pressure	39	32.5%	81	67.5%	120	100.0%		0.325^‡^
Absent	1	100.0%	0	0.0%	1	100.0%	(2)^§^	
Present	38	31.9%	81	68.1%	119	100.0%		
Assumption of a three-point position	39	32.5%	81	67.5%	120	100.0%		0.172^‡^
Absent	39	33.9%	76	66.1%	115	100.0%	1.00	
Present	0	0.0%	5	100.0%	5	100.0%	(1)^†^	
Tachypnea	39	32.5%	81	67.5%	120	100.0%		<0.001*
Absent	39	40.2%	58	59.8%	97	100.0%	1.00	
Present	0	0.0%	23	100.0%	23	100.0%	(1)^†^	
Cough	39	32.5%	81	67.5%	120	100.0%		0.001*
Absent	39	40.6%	57	59.4%	96	100.0%	1.00	
Present	0	0.0%	24	100.0%	24	100.0%	(1)^†^	
Use of accessory muscles to breathe	39	32.5%	81	67.5%	120	100.0%		<0.001*
Absent	39	39.0%	61	61.0%	100	100.0%	1.00	
Present	0	0.0%	20	100.0%	20	100.0%	(1)^†^	
Decreased minute ventilation	39	32.5%	81	67.5%	120	100.0%		1.000^‡^
Absent	39	33.1%	79	66.9%	118	100.0%	1.00	
Present	0	0.0%	2	100.0%	2	100.0%	(1)^†^	

*p-value = descriptive level of Chi-Square; ^†^(1) OR not presented = absence of negative IBP cases in the presence of the defining characteristic; ^‡^Fisher’s exact test; ^§^(2) OR not presented = absence of positive IBP cases in the absence of the defining characteristic

Table 2 shows the sensitivity, specificity and positive and negative related values of DC of patients with IBP. It is notable that only the defining characteristic reduced vesicular murmurs showed high sensitivity (92.6%), high specificity (97.4%) and PPV and NPV above 86%. Auscultation with adventitious sounds also showed high sensitivity (71.6%), specificity (100.0%) and PPV (100.0%), but moderate NPV (62.9%). The other characteristics, except abnormal breathing pattern and decreased inspiratory pressure, presented high specificity, but low sensitivity. An inverse pattern was observed for abnormal breathing pattern and maximal inspiratory pressure.

**Table 2:** Sensitivity, specificity and positive and negative related values of the defining characteristics of Ineffective Breathing Pattern. Rio Branco, AC, Brazil, 2015-2016

**Defining characteristics**	**Sensitivity (%)**	**Specificity (%)**	**Related value (%)**	

			**Positive**	**Negative**
Reduced vesicular murmurs	92.6 (84.6 - 97.2)	97.4 (86.5 - 99.9)	98.7 (92.9-100.0)	86.4 (72.6 - 94.8)
Auscultation with adventitious sounds	71.6 (60.5 - 81.1)	100.0 (91.0 - 100.0)	100 (93.8-100.0)	62.9 (49.7 - 74.8)
Abnormal breathing pattern	97.5 (91.4 - 99.7)	7.7 (1.6 - 20.9)	68.7 (59.4-77.0)	60.0 (14.7 - 94.7)
Decreased inspiratory pressure	100.0 (95.5- 100)	2.6 (0.1 - 13.5)	68.1 (58.9-76.3)	100.0 (2.5-100.0)
Decreased expiratory pressure	100.0 (-)*	0.0 (-)*	67.5 (-)	-
Changes in respiratory depth	40.7 (29.9 - 52.2)	100.0 (91.0 - 100.0)	100 (89.4 -100.0)	44.8 (34.1 - 55.9)
Dyspnea	33.3 (23.2 - 44.7)	100.0 (91.0 - 100.0)	100.0 (87.2-100.0)	41.9 (31.8 - 52.6)
Cough	29.6 (20.0 - 40.8)	100.0 (91.0 - 100.0)	100 (85.8 - 100.0)	40.6 (30.7 - 51.1)
Tachypnea	28.4 (18.9 - 39.5)	100.0 (91.0 - 100.0)	100.0 (85.2-100.0)	40.2 (30.4 - 50.7)
Use of accessory muscles to breathe	24.7 (15.8 - 35.5)	100.0 (91.0 - 100.0)	100.0 (83.2-100.0)	39.0 (29.4 - 49.3)
Altered chest excursion	9.9 (4.4 - 18.5)	100.0 (91.0 - 100.0)	100.0 (63.1-100.0)	34.8 (26.1 - 44.4)
Increased anteroposterior chest diameter	6.2 (2.0 - 13.8)	100.0 (91.0 - 100.0)	100.0 (47.8-100.0)	33.9 (25.3 - 43.3)
Nose wing beats	6.2 (2.0 - 13.8)	100.0 (91.0 - 100.0)	100.0 (47.8-100.0)	33.9 (25.3 - 43.3)
Assumption of a three-point position	6.2 (2.0 - 13.8)	100.0 (91.0 - 100.0)	100.0 (47.8-100.0)	33.9 (25.3 - 43.3)
Decreased minute ventilation	2.5 (0.3 - 8.6)	100.0 (91.0 - 100.0)	100.0 (15.8-100.0)	33.1 (24.7 - 42.3)
Pursed-lip breathing	2.5 (0.3 - 8.6)	100.0 (91.0 - 100.0)	100.0 (15.8-100.0)	33.1 (24.7 - 42.3)
Orthopnea	1.2 (0.0 - 6.7)	100.0 (91.0 - 100.0)	100.0 (2.5 - 100.0)	32.8 (24.4 - 42.0)
Prolonged expiratory phase	1.2 (0.0 - 6.7)	100.0 (91.0 - 100.0)	100.0 (2.5 - 100.0)	32.8 (24.4 - 42.0)
Bradypnea	0.0 (-)*	100.0 (-)*	-	32.5 (-)*

*(-) = it was not possible to calculate because the defining characteristic did not present one of the levels

Table 3 shows the RF of the nursing diagnosis IBP in both groups, with and without the IBP nursing diagnosis. The RF that were associated with IBP were group of diseases, fatigue, obesity and bronchial secretion. Thus, patients with fatigue, obesity and bronchial secretion had higher percentages of IBP compared to those without these conditions. On the other hand, patients diagnosed with cardiocirculatory and respiratory diseases and other groups of diseases presented lower percentages of IBP in comparison to those diagnosed with trauma. The mean age of patients with IBP was higher than those without IBP. The related conditions musculoskeletal damage, chest wall deformity, bone deformity and hypoventilation syndrome were presented in 67.5% of the patients and, not be statistically significant between the groups (p-value>0.05).

**Table 3:** Related factors according to presence or absence of the Nursing Diagnosis Ineffective Breathing Pattern. Rio Branco, AC, Brazil, 2015-2016

	**Ineffective Breathing Pattern**				**Total**		**ODDS RATIO**	**p-value**

	**Absent**		**Present**					

	**n**	**%**	**n**	**%**	**N**	**%**		
Anxiety	39	32.5%	81	67.5%	120	100.0%		0.272*
Absent	38	33.9%	74	66.1%	112	100.0%	1.00	
Present	1	12.5%	7	87.5%	8	100.0%	3.59	
Group of diseases	39	32.5%	81	67.5%	120	100.0%		0.008^†^
Trauma	6	16.7%	30	83.3%	36	100.0%	1.00	
Cardiocirculatory	13	37.1%	22	62.9%	35	100.0%	0.34	
Respiratory	5	22.7%	17	77.3%	22	100.0%	0.68	
Others	15	55.6%	12	44.4%	27	100.0%	0.16	
Pain	39	32.5%	81	67.5%	120	100.0%		0.058*
Absent	38	35.5%	69	64.5%	107	100.0%	1.00	
Present	1	7.7%	12	92.3%	13	100.0%	6.61	
Fatigue	39	32.5%	81	67.5%	120	100.0%		<0.001^†^
Absent	38	48.7%	40	51.3%	78	100.0%	1.00	
Present	1	2.4%	41	97.6%	42	100.0%	38.95	
Respiratory muscle fatigue	39	32.5%	81	67.5%	120	100.0%		1.000*
Absent	39	33.1%	79	66.9%	118	100.0%	1.00	
Present	0	0.0%	2	100.0%	2	100.0%	(1)^†^	
Hyperventilation	39	32.5%	81	67.5%	120	100.0%		0.172*
Absent	39	33.9%	76	66.1%	115	100.0%	1.00	
Present	0	0.0%	5	100.0%	5	100.0%	(1)^†^	
Obesity	39	32.5%	81	67.5%	120	100.0%		0.019*
Absent	35	38.0%	57	62.0%	92	100.0%	1.00	
Present	4	14.3%	24	85.7%	28	100.0%	3.68	
Position of the body that prevents lung expansion	39	32.5%	81	67.5%	120	100.0%		0.550*
Absent	39	33.3%	78	66.7%	117	100.0%	1.00	
Present	0	0.0%	3	100.0%	3	100.0%	(1)^†^	
Bronchial secretion	39	32.5%	81	67.5%	120	100.0%		0.016*
Absent	39	35.8%	70	64.2%	109	100.0%	1.00	
Present	0	0.0%	11	100.0%	11	100.0%	(1)^†^	
Smoking	39	32.5%	81	67.5%	120	100.0%		0.155*
No	27	38.0%	44	62.0%	71	100.0%	1.00	
Yes	8	32.0%	17	68.0%	25	100.0%	1.30	
Ex-smoker	4	16.7%	20	83.3%	24	100.0%	3.07	

*p-value = descriptive level of Fisher’s exact test or Chi-Square; ^†^(1) OR not presented = absence of negative Ineffective Breathing Pattern cases in the presence of the related factor

Table 4 shows the related univariate and multivariate logistic regression models. Patients with fatigue were observed to present a chance of having IBP 61.96 times greater than those without fatigue. On the other hand, it was observed that patients with cardiocirculatory diseases and with other types of diseases were, respectively, 93% and 85% less likely to have IBP than those diagnosed with trauma. It was also noted that with the increase of one year of age, the chance of IBP increases by 6%.

**Table 4:** Final related univariate and multivariate logistic regression models. Rio Branco, AC, Brazil, 2015-2016

	**Univariate model**		**Final multivariate model**	

	**Gross Odds Ratio (95%)**	p-value*	**Adjusted Odds Ratio (95%)**	p-value*
Anxiety	3.59 (0.43- 30.29)	0.239	-	-
Chest wall deformity	(1)^†^	0.999	-	-
Bone deformity	(2)^‡^	-	-	-
Pain	6.61 (0.83- 52.8)	0.075	-	-
Fatigue	38.95 (5.10- 297.4)	<0.001	61.96 (6.88- 557.74)	<0.001
Respiratory muscle fatigue	(1)^†^	0.999	-	-
Hyperventilation	(1)^†^	0.999	-	-
Obesity	3.68 (1.18- 11.51)	0.025	2.76 (0.64- 11.84)	0.171
Position of the body that prevents lung expansion	(1)^†^	0.999	-	-
Musculoskeletal damage	3.04 (0.35- 26.17)	0.311	-	-
Hypoventilation syndrome	(1)^†^	1.000	-	-
Bronchial secretion	(1)^†^	0.999	-	-
Age (years)	1.03 (1.01- 1.05)	0.009	1.06 (1.02- 1.09)	0.001
Smoking (ref. = no)		0.172		
Yes	1.30 (0.5- 3.43)	0.591		
Ex-smoker	3.07 (0.95- 9.94)	0.062		
Medical diagnosis (ref. = trauma)		0.011		0.013
Cardiocirculatory	0.34 (0.11- 1.03)	0.056	0.07 (0.01- 0.38)	0.002
Respiratory	0.68 (0.18- 2.56)	0.569	0.23 (0.04- 1.30)	0.096
Others	0.16 (0.05- 0.51)	0.002	0.15 (0.04- 0.62)	0.009

*Hosmer and Leme show test for goodness of fit of the model (p = 0.649); ^†^(1) = absence of negative Ineffective Breathing Pattern cases in the presence of the related factor; ^‡^(2) = absence of one of the levels of the related factor

**Figure 1:**
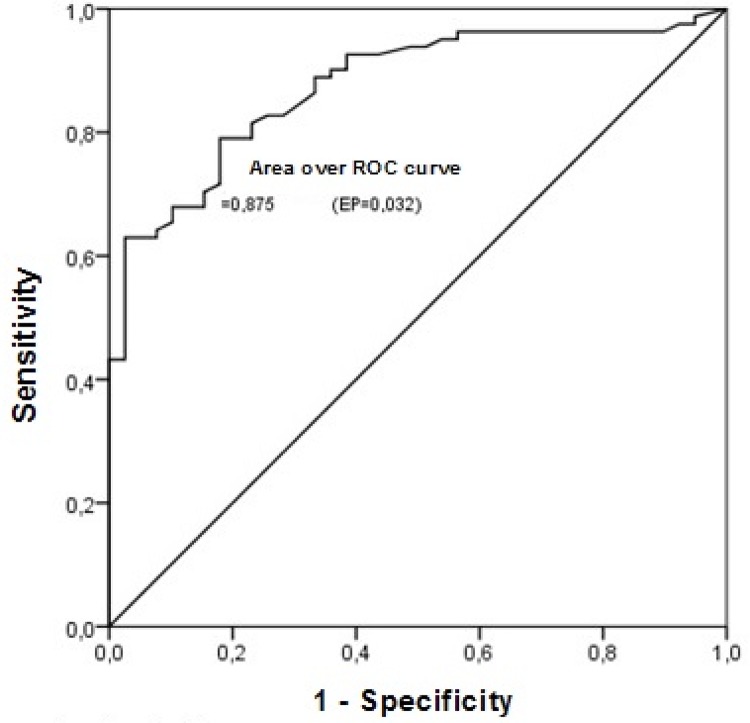
ROC curve for Ineffective Breathing Pattern. Rio Branco, AC, Brazil, 2015-2016

The final model, given the information of the related factors, allows to estimate the probability of a patient to present IBP. Using the ROC curve, a good related capacity of the probabilities of occurrence of IBP estimated by the final model (area under the ROC curve 0.875) with high sensitivity (82.72%) and specificity (74.36%) is observed (Figure 1).

## Discussion

The related factors of the nursing diagnosis IBP in the studied ICU were fatigue, age, and group of diseases (cardiocirculatory diseases, trauma and other diagnoses). The development of fatigue is an important and common complication in many patients admitted to ICUs and its incidence may range from 30% to 60% in these patients^11,24^. Fatigue has been investigated in many studies because of its high prevalence and the damage caused to the patients’ quality of life^18,25-27^. Besides previous co-morbidities, several factors may contribute to fatigue, including systemic inflammation, use of some medications such as corticoids, sedatives and neuromuscular blockers, malnutrition, hyperosmolarity, parenteral nutrition, cardiopathies and prolonged immobility, common conditions in the ICU^25-26^. Fatigue can be present in patients with diverse pathologies, such as heart, lung, hematological, and oncological diseases, as well as in patients presenting pain, malnutrition and psychological manifestations such as anxiety and depression, which also corroborates the other RF found in this study, that is, group of diseases^27^.

Other factors that may contribute to the onset of fatigue are age and number of comorbidities^17^. Regarding the number of morbidities, studies have shown an association between greater number of morbidities and greater perception of fatigue^28-29^. In the elderly, fatigue occurs due to changes in the body as a whole and in the cardiopulmonary system, in which reduction of oxygen uptake, reduction of respiratory muscle strength, and increase of vascular resistance are observed. In the muscular system, there is a decrease in muscle strength and flexibility, resulting in fatigue, which affects simple activities of daily life of the elderly^30^.

A recent study, also performed in an ICU in the city of Ribeirão Preto, SP, Brazil, with 626 adult patients, showed that the RF fatigue presented greater sensitivity for the IBP diagnosis^23^. The authors emphasize that IBP patients present DC related to ventilatory dysfunction and, if not treated adequately, this diagnosis may evolve to the diagnosis of impaired spontaneous ventilation (ISV), characterizing a worse prognosis of the patient^23^.

Old age was the second predictor of IBP in this study. Ageing is characterized by a chronic decrease in the functions of the organic system, leaving the elderly susceptible to diseases, with risk to trigger the IBP diagnosis^2^. Ageing leads to physiological changes such as compromised gas exchange efficiency, reduced pulmonary compliance, decreased respiratory muscle strength, and decreased oxygen transport to tissues, resulting in decreased cardiac output, body mass, alveolar volume and ventilation/perfusion ratio, which may lead to the emergence of the Nursing diagnosis IBP^20^.

In this sense, it is up to nurses to recognize these peculiarities and alterations during the physical examination and to select interventions that improve the respiratory state within the expected for the age. A cross-sectional study conducted in Rio Grande do Sul, Brazil, identified that almost half of the elderly (42.0%) had IBP^31^. In this study, 86.7% of elderly patients presented IBP. It is also worth mentioning that this group of patients is more vulnerable to influenza due to the higher prevalence of chronic degenerative diseases and immunological deterioration, which may cause breathing changes and the manifestation of IBP^31-34^.

The third related factor of IBP was group of diseases (trauma, cardiocirculatory diseases, and other diagnoses). External causes, as an important cause of traumas, represented by traffic accidents, represent a serious public health problem in Brazil and are responsible for high morbidity and mortality, disability rates, and sequelae, not to mention considerable economic cost^35^. A study that analyzed 406 trauma victims in the city of São Paulo, SP, Brazil, identified a prevalence of 82.8% of patients with IBP^5^.

Another study performed in the urgency and emergency unit of a large public hospital in southern Brazil identified a prevalence of 51.2% of IBP in patients who had been victims of multiple traumas, the main ones being pain, skeletal muscle damage, hyperventilation and neuromuscular dysfunction, and the main DC was tachypnea and bradypnea^36^. The presence of these clinical indicators occurs due to the changes in pulmonary expansion following the alteration of the chest cavity, besides hypovolemia following hemorrhage and hypoxia caused by traumatic lesions. Thus, the control of cerebral oxygenation and the supply of oxygen to the other organs of the body are fundamental in the care of these patients, besides the control of bleeding. The lack of attention to respiratory care may lead patients to develop IBP^36-37^.

In relation to the group of diseases related to the cardiocirculatory system, it is known that patients with these comorbidities may present cardiac decompensation with consequent hemodynamic changes, giving rise to the IBP diagnosis. Patients with left heart failure, for example, may present signs and symptoms of pulmonary congestion due to left ventricular failure, evidencing the nursing diagnosis IBP. Moreover, patients with acute coronary syndrome have chest pain as their main symptom. Anginal pain triggers manifestations of the sympathetic system that cause an increase in heart rate and breathing, altering the breathing mechanics regarding depth, number of incursions per minute. Without intervention, this will lead to respiratory muscle fatigue and consequent IBP diagnosis^17^.

Thus, in the analysis of the final logistic regression model of the related factors of IBP, the curve presented a good related capacity of the probability of occurrence of IBP (ROC curve 0.875), with high sensitivity and specificity to identify this nursing diagnosis.

Other related factors (obesity and bronchial secretion), although not identified as predictors of IBP in our study, have been also associated with this diagnosis. It was also observed that the DC changes in respiratory depth, auscultation with adventitious sounds, dyspnea, reduced vesicular murmurs, tachypnea, cough and use of accessory respiratory muscles were also associated with IBP^12-14,19^.

In obese individuals, IBP diagnosis is detected by the reduced lung volume and capacity in these patients. Excessive adipose tissue also causes mechanical compression of the diaphragm, resulting in restrictive respiratory insufficiency, decreased pulmonary compliance and increased pulmonary resistance, which consequently increases respiratory work and oxygen, resulting in the IBP diagnosis^38-39^.

The RF bronchial secretion possibly related to the Nursing diagnosis IBP due to the narrowing of the lumen as consequence of the exacerbated production of secretions and also due to the inability of intensive care patients to expel secretions spontaneously from the respiratory tract, leading to respiratory difficulty and to the IBP diagnosis^12^.

The DC dyspnea, tachypnea, changes in respiratory depth and use of accessory muscles to breathe are very common alterations among patients with IBP. This is due to respiratory muscle weakness and non-resolution of the underlying problem that led in the first place to respiratory decompensation. Tachypnea is the result of pulmonary hyperventilation, which develops as an adaptive compensation attempt^18^. The failure of this compensatory mechanism and the imbalance between the demand and the supply of oxygen favor the appearance of the IBP diagnosis^40-41^. The use of the accessory musculature demonstrates the attempt to re-establish a normal breathing pattern. A study carried out in the city of Fortaleza, CE, Brazil, identified that the use of the accessory musculature brings a seven-fold higher chance of having the IBP diagnosis^15^.

Cough is a symptom of a wide variety of pulmonary and extra-pulmonary diseases, and is very prevalent in the population, has a negative social impact, non-tolerated at work and family contexts, besides generating a great cost in terms of exams and medications. The major causes of cough are viral infections of the upper airways (common cold), lower airways (acute tracheobronchitis), acute sinusitis, exposure to allergens and irritants, and exacerbations of chronic diseases such as asthma, chronic obstructive pulmonary disease (COPD) and rhinosinusitis^42^, which can cause changes in pulmonary ventilation, leading the individual to present the IBP diagnosis. A cross-sectional study carried out in Fortaleza, CE, Brazil, showed that the IBP diagnosis was the most prevalent and the most common DC were adventitious respiratory sounds and cough^43^. Adventitious respiratory sounds are detected in pulmonary auscultation and are common in patients with respiratory changes in ICUs^12^.

When assessing the specificity, sensitivity and positive and negative related values of DC and RF, it was observed that the DC reduced vesicular murmurs had an association and an excellent measure of accuracy, presenting sensitivity, specificity, positive related value and negative high values for the nursing diagnosis IBP.

Despite its importance, the DC reduced vesicular murmurs is not part of the NANDA-I classification for this nursing diagnosis. Vesicular murmurs are normal sounds auscultated in the lungs and their decrease is pathological and may indicate the presence of atelectasis and even decreased lung expansion^44^. Atelectasis is a respiratory complication caused by the obstruction of a bronchus, or lung, by secretion or solid bodies that prevent the flow of air and lead to a decrease in the number of alveoli worked^44^. When there is complete obstruction in a bronchus that supplies air to a normally ventilated region of the lung parenchyma, the gas in the alveoli distal to the obstruction is absorbed into the pulmonary circulation. Once all the alveolar gas is absorbed into the circulation, the alveoli, now without gas, collapse, generating a decrease in vesicular murmurs and causing changes in the respiratory ventilation and ineffective breathing pattern^44^.

The results of this study showed that there are related factors for the nursing diagnosis IBP and nursing interventions and early targeting should be performed in the case of patients with fatigue, advanced age, with problems such as trauma, cardiocirculatory diseases and other diseases.

As a positive factor, this research used a large sample of critical patients, an objective measurement, manovacuometry, and contributed with new DC and RF for the Nursing diagnosis IBP, which will provide the improvement of the NANDA International, Inc. classification of nursing diagnoses, making it possible a more accurate nursing education, besides bringing evidence to the clinical practice of diagnosing in nursing.

## Conclusion

Related factors of IBP were fatigue, old age, trauma, cardiocirculatory diseases and other diseases. When analyzing the final model through the ROC curve, it was observed that the model had a good related capacity for IBP, associated to high specificity and sensitivity. The DC reduced murmurs presented high sensitivity, specificity and related and negative values for IBP, demonstrating its importance in the identification of this nursing diagnosis.

## References

[1] Sarkar M, Madabhavi I, Niranjan N, Dogra M (2015). Auscultation of the respiratory system. Ann Thorac Med.

[2] Wuytack F, Meskell P, Conway A, McDaid F, Santesso N, Hickey FG (2017). The effectiveness of physiologically based early warning or track
and trigger systems after triage in adult patients presenting to emergency
departments: a systematic review. BMC Emerg Med.

[3] Kim MJ, Larson JL (1987). Ineffective airway clearance and ineffective breathing patterns:
Theorical and research base for nursing diagnosis. Nurs Clin North Am.

[4] Herdman TH, Kamitsuru S (2018). Diagnósticos de Enfermagem da NANDA: definições e classificação
2018-2020/ [NANDA Internacional].

[5] Sallum AMC, Santos JLF, Lima FD (2012). Nursing diagnoses in trauma victims with fatal outcomes in the
emergency scenario. Rev. Latino-Am. Enfermagem.

[6] Okuno MFP, Costa N, Lopes MCBT, Campanharo CRV, Batista REA (2012). The most used nursing diagnoses at an emergency
service. Acta Paul Enferm.

[7] Galdeano LE, Rossi LAR, Pezzuto TM (2004). Nursing diagnosis of patients in the preoperatory period of
cardiac surgery. Rev Esc Enferm USP.

[8] Gordon M, Hiltunen E (1995). High frequency: treatment priority Nursing diagnoses in critical
care. Nurs Diagn.

[9] Araújo DS, Freire AF, Mendonça JKS, Bettencourt ARC, Amaral TLM, Prado PR (2015). Construction and validation of a systematization instrument for
nursing in intensive care. Rev Rene.

[10] Black LF, Hyatt RE (1969). Maximal respiratory pressures: normal values and relationship to
age and sex. Am Rev Respir Dis.

[11] Souza RB (2002). Sociedade Brasileira de Pneumologia e Tisiologia. Diretrizes para
teste de função pulmonar. Pressões respiratórias estáticas
máximas. Jornal Bras Pneumol.

[12] Prado PR, Bettencourt ARC, Lopes JL (2018). Defining characteristics and related factors of nursing diagnosis
ineffective breathing pattern: na integrative literature
review. Rev Bras Enferm.

[13] Avena M, Pedreira MLG, Gutiérrez MGR (2014). Conceptual validation of the defining characteristics of
respiratory nursing diagnoses in neonates. Acta Paul Enferm.

[14] Cavalcante JCBC, Mendes LC, Lopes MVO, Lima LH (2010). Clinical indicators of ineffective breathing pattern in children
with asthma. Rev RENE.

[15] Silva VM, Araujo TL, Lopes MVO (2006). Evolution of nursing diagnoses for children with congenital heart
disease. Rev. Latino-Am. Enfermagem.

[16] Bertoncello KCG, Cavalcanti CDK, Ilha P (2013). Real diagnoses and nursing intervention proposals for multiple
trauma victims. Rev Eletron Enferm.

[17] Canto DF, Almeida MA (2013). Nursing outcomes for ineffective breathing patterns and impaired
spontaneous ventilation in intensive care. Rev Gaúcha Enferm.

[18] Santos NA, Cavalcante TF, Lopes MVO, Gomes EB, Oliveira CJ (2015). Profile of nursing diagnoses in patients with respiratory
disorders. Invest Educ Enferm.

[19] Mota DDCF, Cruz DALM, Pime CAM (2005). Fatigue: a concept analyses. Acta Paul Enferm.

[20] Ferreira EVM (2015). Respiratory muscles: myths and secrets. J Bras Pneumol.

[21] Silva GA (2006). Obesity hypoventilation syndrome. Medicina.

[22] Caruso P, Albuquerque ALP, Santana PV, Cardenas LZ, Ferreira JG, Prina E (2015). Diagnostic methods to assess inspiratory and expiratory muscle
strength.

[23] Seganfredo DH, Beltrão BA, Silva VM, Lopes MVO, Castro SMJ, Almeida MA (2017). Analysis of ineffective breathing pattern and impaired
spontaneous ventilation of adults with oxygen therapy Rev. Latino-Am. Enfermagem.

[24] Ali NA, O’Brien JM, Hoffmann SP, Phillips G, Garland A, Finley JC, Midwest Critical Care Consortium (2008). Acquired weakness, handgrip strength, and mortality in critically
ill patients. Am J Respir Crit Care Med.

[25] Maramattom BV, Wijdicks EF (2006). Acute neuromuscular weakness in the intensive care
unit. Critical Care Medicine. Crit Care Med.

[26] Khan J, Harrison TB, Rich MM (2008). Mechanisms of neuromuscular dysfunction in critical
illness. Crit Care Clin.

[27] Truong AD, Fan E, Brower RG, Needham DM (2009). Benchto-beside review: mobilizing patients in the intensive care
unit from pathophysiology to clinical trials. Crit Care.

[28] Latronico N, Herridge M, Hopkins RO, Angus D, Hart N, Hermans G (2017). The ICM research agenda on intensive care unit-acquired
weakness. Intensive Care medicine.

[29] Castell MV, Sânchez M, Julián R, Queipo R, Martín S, Otero A (2013). Frailty prevalence and slow walking speed in persons age 65 and
older: implications for primary care. BMC Fam Pract.

[30] Neri AL, Yassuda MA, Araújo LF, Eulálio MC, Cabral BE, Siqueira MEC (2013). Methodology and social, demographic, cognitive, and frailty
profiles of community-dwelling elderly from seven Brazilian cities: the
FIBRA Study. Cad Saúde Pública.

[31] Lira LN, Santos SSC, Vidal DAS, Gautério DP, Tomaschewski-Barlem JG, Piexak DR (2015). Nursing diagnosis and prescriptions for hospitalized
elderly. Av Enferm.

[32] Talbot HK (2017). Influenza in older Adults. Infect Dis Clin North Am.

[33] Stockton J, Stephenson I, Fleming D, Zambon M (2002). Human metapneumovirus as a cause of community-acquired
respiratory illness. Emerg Infect Dis.

[34] Boivin G, Abed Y, Pelletier G, Ruel L, Moisan D, Côté S (2002). Virological features and clinical manifestations associated with
human metapneumovirus: a new paramyxovirus responsible for acute
respiratory-tract infections in all age groups. J Infect Dis.

[35] Santos ZM, Oliveira ML (2010). Assessment of knowledge, attitudes and practices of the elderly
about the vaccine against Influenza in a Public Health Unit, Taguatinga,
Federal District, Brazil, 2009. Epidemiol Serv Saúde.

[36] Malvestio MAA, Sousa RMC (2008). Survival after motor vehicle crash: impact of clinical and
prehospital variables. Rev Saúde Pública.

[37] ATLS Subcommittee, American College of Surgeons’ Committee on Trauma, International ATLS working group (2013). Adv Trauma Life Support (ATLS^®^): the ninth
edition. J Trauma Acute Care Surg.

[38] Dal Sasso GTM, Barra DCC, Paese F, Almeida SRW, Rios GC, Marinho MM (2013). Computerized nursing process: methodology to establish
associations between clinical assessment, diagnosis, interventions, and
outcomes. Rev Esc Enferm USP.

[39] Schmidt M, Demoule A, Polito A, Porchet R, Aboab J, Siami S (2011). Dyspnea in mechanically ventilated critically ill
patients. Crit Care Med.

[40] Goodridge D, Duggleby W, Gjevre J, Rennie D (2009). Exploring the quality of dying of patients with chronic
obstructive pulmonary disease in the intensive care unit: a mixed methods
study. Nurs Crit Care.

[41] Sociedade Brasileira de Pneumologia e Tisiologia (2006). IV Diretrizes brasileiras no manejo da asma. J Bras Pneumol.

[42] Pratter MR, Brightling CE, Boulet LP, Irwin RS (2006). An empiric integrative approach to the management of cough: ACCP
evidence-based clinical practice guidelines. Chest.

[43] Silveira UA, Lima LHO, Lopes MVO (2008). Defined characteristics of the nursing diagnoses ineffective
airway clearance and ineffective breathing pattern in asthmatic
children. Rev RENE.

[44] Cunha CS, Toledo RV (2007). The Performance of the Physiotherapy in the Reversion of the
Atelectasis: A report of a case in the Intensive Therapy
Unit. Cads Unifoa.

